# Calcium Intake in Bone Health: A Focus on Calcium-Rich Mineral Waters

**DOI:** 10.3390/nu10121930

**Published:** 2018-12-05

**Authors:** Letizia Vannucci, Caterina Fossi, Sara Quattrini, Leonardo Guasti, Barbara Pampaloni, Giorgio Gronchi, Francesca Giusti, Cecilia Romagnoli, Luisella Cianferotti, Gemma Marcucci, Maria Luisa Brandi

**Affiliations:** 1 Department of Surgery and Translational Medicine, University of Florence, Viale Pieraccini 6, 50139 Florence, Italy; letizia.vannucci@unifi.it (L.V.); caterina.fossi@unifi.it (C.F.); sara.quattrini@unifi.it (S.Q.); leonardo.guasti@unifi.it (L.G.); barbara.pampaloni@unifi.it (B.P.); francesca.giusti1@alice.it (F.G.); cecilia.romagnoli@unifi.it (C.R.); luisella.cianferotti@unifi.it (L.C.); gemma.marcucci@unifi.it (G.M.); 2 Department of Neurosciences, Psychology, Drug Research, and Child Health (Section of Psychology), University of Florence, Via di San Salvi 12, 50139 Florence, Italy; giorgio.gronchi@gmail.com

**Keywords:** Calcium, mineral water, calcium-rich water, bone metabolism, calcium homeostasis

## Abstract

Calcium is an essential element that plays numerous biological functions in the human body, of which one of the most important is skeleton mineralization. Bone is a mineralized connective tissue in which calcium represents the major component, conferring bone strength and structure. Proper dietary calcium intake is important for bone development and metabolism, and its requirement can vary throughout life. The mineral composition of drinking water is becoming relevant in the modulation of calcium homeostasis. In fact, calcium present in mineral drinking waters is an important quantitative source of calcium intake. This, together with its excellent bioavailability, contributes to the maintenance of the bone health. This article aims to examine studies that assessed the bioavailability of the calcium contained in calcium-rich mineral waters and their impact on bone health, including original data collected in a recent study in humans.

## 1. Introduction

Calcium is one of the most abundant elements in the human body and is a major component of the mineralized tissues where more than 99% of total body calcium is contained. It plays a key role in skeleton mineralization and is required for normal growth, development, and bone strength [1]. Moreover, it has a role in a wide range of biological functions, such as muscle contraction and nerve impulse transmission [2]. Since calcium is fundamental for many essential functions, it is important that its concentration in body fluids is maintained within a physiological range, thanks to the fine regulation by calcitropic hormones [3]. Calcium is an essential element and is, therefore, essential to introduce the recommended quantity through the diet.

In this manuscript, we will review literature focusing on calcium-rich mineral waters as a source of calcium and their effects on bone metabolism. Moreover, a case report will be treated in order to support evidence that calcium-rich mineral water is a valuable nutritional source of highly bioavailable calcium and can significantly contribute to achieving the daily requirement of this element.

## 2. Background

Bone is a complex cellular tissue that contains, by weight, approximately 70% mineral and 30% organic constituents. The mineral phase consists of about 95% hydroxyapatite, Ca_10_(PO_4_)_6_(OH)_2_, a highly organized crystal of calcium and phosphorous, and other ions (such as sodium, magnesium, fluoride, and strontium). The organic phase (osteoid) is composed of 98% collagen fibers, and by a ground substance formed by glycoproteins and proteoglycans [4]. 

Bone is a mineralized connective tissue that exhibits two different types of bone: (1) Cortical bone, which is the compact, thick, and dense layer that forms the outer surface of most bone and the shafts of the long bones; (2) cancellous or trabecular bone, which has the aspect of a sponge and it is found at the end of long bones and within flat bones and vertebrae. The cortical bone has a predominantly structural function, since 80%–90% of its volume is calcified, whereas the role of the trabecular bone is regarded as metabolic and only 15%–25% calcified [5].

Despite its inert appearance, bone is a highly dynamic organ that is continuously remodeled through the concerted actions of bone cells. The normal bone remodeling process occurs due to the coordinated actions of osteoclasts, which are responsible for the resorption of bone, osteoblasts, which secrete osteoid and modulate crystallization of hydroxyapatite, and osteocytes, which are embedded within the mineralized region of bone and are involved in the mechanosensitive function in the internal bone environment, allowing communication among bone cells. Another type of cell, called bone lining cell, is present in the bone, but the function is not completely understood, although these cells seem to play an important role in coupling bone resorption to bone formation [6,7].

Bone remodeling and turnover is a key point for maintaining the structural function of the skeleton and for fracture healing; on the other hand, an imbalance of bone resorption and formation results in several bone diseases. For example, excessive resorption by osteoclasts without the corresponding amount of newly formed bone by osteoblasts contributes to bone loss and osteoporosis, an age-related disease, characterized by low bone mass and deterioration of bone tissue with a consequent increase in fragility and susceptibility to fractures, whereas the contrary characterizes osteopetrosis [8,9,10].

Calcium is an essential element that plays numerous biological functions in the body, and one of the most important is the skeletal mineralization. Calcium is the major component of the bone, where it is present at more than 99% as calcium-phosphate complexes, and provides the skeleton strength and structure, making the bone a metabolic reservoir to maintain the intra- and extra-cellular calcium pool. The remaining part is present in blood, in extracellular fluids, in muscles and other tissues, where it is responsible for mediating muscle contraction, vascular contraction and vasodilatation, nerve impulse transmission, and intra and extracellular signaling [1,2].

The concentration of serum ionized calcium is tightly maintained at the physiological range between 1.10–1.35 mM in healthy subjects, by the action of calciotropic hormones: parathyroid hormone (PTH), 1,25-dihydroxyvitamin D [1,25(OH)_2_D], Fibroblast Growth Factor 23 (FGF23), and calcitonin. A decrease in serum calcium concentration increases from the parathyroid glands, the secretion of PTH which acts on the PTH receptor in the bone to release calcium and in the kidneys to increase tubular calcium reabsorption, thereby reducing urinary calcium excretion. Moreover, in the kidneys, PTH stimulates the secretion of 1,25(OH)_2_D, which activates the vitamin D receptor (VDR) in gut and, therefore, it increases active calcium absorption. Increases in serum calcium are reversed by calcitonin, which is a hormone secreted by the thyroid gland and through a negative feedback by 1,25(OH)_2_D on PTH secretion. FGF23 controls the phosphate serum levels and, therefore, indirectly the calcemia. 

The reciprocal feedback mechanisms among the calciotropic hormones are key in maintaining calcium homeostasis in healthy individuals [3,11,12].

It is widely recognized that bone mass and density are determined by various concurrent factors, such as genetics, hormones, physical activity, and certainly, nutrition. While genetic factors have a critical role in growth and peak bone development, an adequate intake of bone nutrients represents the main factor for the full expression of a given genetic potential and for bone maintenance during adulthood [13,14]. Among the various nutrients, calcium and vitamin D have proven their efficacy for normal bone growth and development in children and adolescents and for the maintenance of bone mineral loss in postmenopausal women [15].

An optimal calcium intake is necessary for bone health at all stages of life. Dietary requirements for calcium are determined by the need for bone development and bone maintenance, which vary throughout life, being higher during childhood and adolescence, during pregnancy and lactation, and in the elderly.

Recommended Dietary Allowance for Calcium varies between 700–1200 mg/day throughout life, as stated both at the international level by the United States Department of Agriculture (USDA) (Table 1) [16], and at the Italian level by the Reference Levels of Nutrients and energy intake for the Italian population (LARN, Livelli di Assunzione di Riferimento di Nutrienti ed energia per la popolazione italiana) [17].

Even though dairy products are the most recognized dietary sources of calcium, natural mineral waters are also a potential important source. Water is the main constituent of the human body and is involved in many bodily functions, including being the carrier of nutrients to reach biological fluids and also the main vehicle to eliminate waste and toxins [18]. Since the body lacks the water storage, a constant supply of fluids is needed to maintain water balance. According to LARN for water, a daily intake of 1.2 to 2.5 L of water should be guaranteed, because good hydration is essential to maintain body water equilibrium, although needs may vary among people, depending on age, physical activity, personal circumstances, and weather conditions [17,19]. The USDA reports that water intake from food, beverages, and drinking water should be 2.7 L per day in women aged 19–50 years [20].

## 3. Calcium-Rich Mineral Waters and Bone Metabolism

The 2009/54/EC directive classified natural mineral waters based on the mineral content, indicating “Water with Calcium” if calcium content is >150 mg/L [21]. High-calcium mineral waters could therefore be recommended to provide both a dietary calcium source and adequate hydration, while being calorie free. 

Since the 1990s, studies have been carried out to assess the bioavailability of calcium contained in calcium-rich mineral water when compared with that of calcium taken with dairy products [22,23,24,25,26,27,28,29,30].

In most studies, calcium absorption was directly measured using the tracer technique with isotopes [22,23,24,25,26,27], whereas other authors used indirect measurements, such as PTH serum concentration [30], urinary [28,29], and serum calcium [28]. While the number of these studies is limited, and generally, small groups of participants were recruited, their convergent results led to a single concordant conclusion: the bioavailability of calcium from calcium-rich mineral waters is equivalent to [28,31,32], or possibly higher than [24], that of calcium contained in milk and dairy products.

Particularly, Bacciottini et al. measured the bioavailability of the calcium contained in a high-calcium mineral water in 27 healthy subjects, comparing it with milk, demonstrating that calcium from mineral water and from milk are similarly bioavailable [22]. Superimposable results were reported in a study by Heaney and Dowell, which compared the bioavailability of calcium from a calcium-rich water and milk in 18 healthy women [23]. 

Following these two pioneering studies, the role of calcic mineral waters on mineral and bone metabolism has been studied, even if in a relatively small number of subjects. The majority of the studies demonstrated that mineral waters rich in calcium have a positive impact on both bone biomarkers and densitometric parameters [33,34,35,36,37,38,39,40]. 

Results of a randomized, placebo-controlled, double-blind trial, conducted by Meunier et al. in 152 postmenopausal healthy women with a low dietary calcium intake (below 700 mg/day), showed that the consumption of a sulphate-rich mineral water with a high calcium content (596 mg/L) reduced circulating PTH and bone turnover markers [35]. 

Acute effects on bone metabolism within four hours from the ingestion of a calcium-rich water have been evaluated in 12 healthy young men in a study by Guillemant et al., showing that the intake of 0.5 L of water containing 172 mg calcium led to a significant transient reduction in serum PTH, with a nadir at one hour from water intake, and a progressively significant reduction in the bone resorption marker type 1 collagen cross-linked C-telopeptide (CTX) [40].

A study by Cepollaro et al., conducted in 45 early postmenopausal women, showed that after about one year of consumption of either calcium-rich or low-content calcium mineral water, the group of women with low calcium intake had a significant decrease in bone mineral density (BMD) of the wrist [37]. 

Costi et al. analyzed a sample of 255 pre- and post-menopausal women. They found that a regular intake of calcium-rich mineral water significantly contributed to maintain vertebral BMD, particularly after menopause [38]. 

Aptel et al. collected data from a large sample of 4434 women within the EPIDemiologie de l’OSteoporose (EPIDOS) trial, a French prospective observational multicenter cohort study of osteoporosis epidemiology, observing that an increase of 100 mg per day of calcium from drinking water was associated with a 0.5% increase in femoral neck BMD in women older than 75 years [39]. 

Table 2 summarizes the main characteristics of the clinical studies that have evaluated calcium bioavailability from mineral water and the effects of this ion on mineral and bone metabolism [22,23,24,25,26,27,28,29,35,36,37,38,39,40]. Overall, these studies support the role of calcic mineral waters as an important source of dietary calcium, that should be considered in order to obtain the recommended daily calcium intake, especially in cases of lactose intolerance. 

## 4. Case Report

We also performed a study investigating the relationship between calcium from calcium-rich mineral water and bone metabolism. Particularly, the aim of our study was to evaluate the effects of calcium from a calcium-rich mineral water on mineral and bone metabolism in a sample of premenopausal healthy women through the measurement of serum and urinary biomarkers. 

### 4.1. Subjects and Methods 

Thirty premenopausal women in good health, with regular menstrual cycles, were recruited to our longitudinal study that lasted nine weeks. 

As daily dietary calcium intake of each participant was not to exceed 700 mg [17], a specific web tool questionnaire to evaluate calcium intake, designed by the International Osteoporosis Foundation (IOF), was administered to the women by trained nutritionists. The questionnaire consisted of 78 food items including: milk and dairy products, fruits, vegetables, legumes, cereals, meats, fish, eggs, nuts, supplements, and cooked dishes. Subjects were asked to report consumption frequency and portion size assessment [41]. If necessary, dietary recommendations were provided to the participants. 

In order to increase adherence and respect of the diet, the women enrolled in the study were selected from nutritionists, dieticians, and/or students from Dietician School at the University Hospital of Florence.

The exclusion criteria included: pregnancy and breastfeeding, body mass index (BMI) < 20 or ≥ 30 kg/m^2^, history of alcohol or drug abuse, current cancer or cancer diagnosed in the last five years, treatments potentially able to affect calcium metabolism (corticosteroids, diuretics, etc.), diseases requiring therapies potentially interfering with bone metabolism (rheumatoid arthritis, kidney failure, hypertension), fragility fractures, and enrolment in other clinical trials during the previous three months.

At the time of enrolment in the study (referred to as visit 1 (V1)), each participant underwent medical history and examination. 

**Figure 1:**
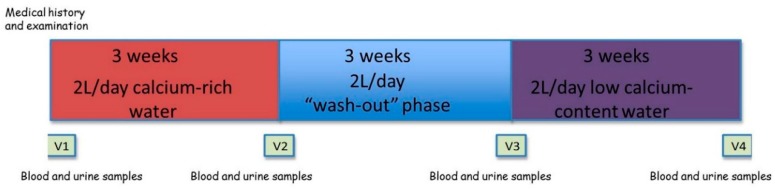
Schematic representation of the study design and procedures. V1: baseline medium-mineral calcium-rich water. V2: +3 weeks of calcium-rich water. V3: baseline low mineralized and low calcium-content water. V4: +3 weeks of low calcium-content water.

The entire study group was assigned to the consumption of a medium-mineral calcium-rich water, marketed under the name of Uliveto, for three weeks; for the next three weeks, each participant returned to drinking the water normally taken before the enrolment in the present study (“wash-out” phase); finally, for the last three weeks, each participant took a low mineralized water with low calcium content, named water B. Daily water consumption was two liters for the entire duration of the study (Figure 1).

The compositions of the Uliveto water and B water are shown in Table 3.

Laboratory parameters related to mineral and bone metabolism were evaluated four times for each participant, particularly at baseline at the time of enrolment in the study (V1), and subsequently every three weeks, specifically at the end of each period of consumption of Uliveto and B waters (referred to as visit 2 (V2) and visit 4 (V4), respectively), and at the end of the “wash-out” phase (referred to as visit 3 (V3)) (Figure 1).

The following serum parameters were evaluated: calcium (mg/dL), phosphate (mg/dL), magnesium (mg/dL), total proteins (g/dL), PTH (pmol/L), 25(OH)vitaminD (ng/mL), bone alkaline phosphatase (BALP) (mcg/L), and beta cross laps (beta-CTX) (ng/mL). Urinary calcium and phosphate excretion were also evaluated by 24 h urine collection (mg/24 h). Complete urine test of fasting morning urine was also performed.

The study was approved by the Institutional Review Board (Comitato Etico Area Vasta Centro, AOUC, Florence, Italy), and informed consent was obtained from all participants included in the study.

### 4.2. Statistical Analysis

Data were expressed as mean ± SD. The comparison between values of biochemical parameters was performed by Student’s *t*-test. Statistical significance was considered as *p* < 0.05. All collected data were subjected to a preliminary verification with respect to the following criteria: Presence of outlier; undetectable values; significantly high values; and erratically performed 24 h urine collection. The term “outlier” is not employed to refer to a statistical outlier (i.e., an observation that is three standard deviations away from the sample mean or similar). Instead, we used the term outlier to refer to abnormal values likely resulting from urinary sample collection that was not reliable. The subjects considered outliers are those whose urinary collection was clearly proven to have not been performed as indicated and this is a criterion we have to use in an observational analysis. According to this verification, all the values of 24 h urinary calcium (V1, V2, V3, and V4) of 10 participants and single values of 24 h urinary calcium (V1; V1, and V2; V3, and V4) of three participants were excluded from statistical analysis.

### 4.3. Results 

Thirty women aged 22 to 45 years were enrolled in the study. Three women voluntarily withdrew from the study, whereas the remaining twenty-seven completed it.

The subjects were enrolled on the basis of their low dietary calcium intake (not exceeding 700 mg/day).

No significant differences emerged between values of the evaluated serum parameters (calcium, phosphate, magnesium, total proteins, PTH, 25(OH)vitaminD, BALP, beta-CTX) and urinary parameter (24 h urinary phosphate) of mineral and bone metabolism. Particularly, no significant difference was found between the mean values of each laboratory parameter measured at V1 and the mean value of the same parameter measured at V2, nor between the mean values of each laboratory parameter measured at V3 and the mean values of the same parameter measured at V4. Biochemical characteristics of mineral and bone metabolism of the entire study group (*n* = 27) are shown in Table 4. 

A significant difference was found between values of 24-h urinary calcium. Particularly, after excluding urinary calcium values according to the preliminary verification, we observed that the mean value of urinary calcium at baseline, before starting to take the calcium-rich water (V1), was 126.33 ± 79.78 mg/24 h, whereas the mean value of urinary calcium at the end of the 3 week period of intake of the calcium-rich mineral water (V2) was 164.27 ± 133.43 mg/24 h. A statistically significant difference was observed between these two mean values of urinary calcium (*p* = 0.034). Specifically, a significant increase of 24-h urinary calcium value, which remained within the normal range, was found to be associated with the consumption of the calcium-rich mineral water.

**Figure 2:**
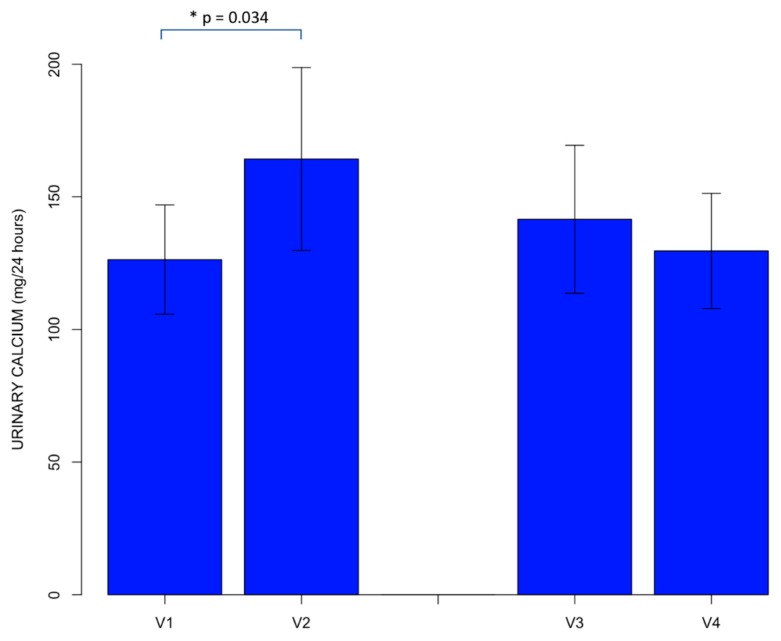
Comparisons between the mean value of 24 h urinary calcium at baseline, before consumption of the calcium-rich water (V1), and the mean value of 24 h urinary calcium after the 3 week period of intake of the calcium-rich water (V2), and between the mean value of 24-h urinary calcium under basal conditions, at the end of the “wash-out” phase, before starting intake of water B (V3), and the mean value of 24 h urinary calcium at the end of the 3 week period of intake of water B (V4), after excluding urinary calcium values according to the preliminary verification. The error bars indicate the standard error of mean. * = statistical significance.

No statistically significant difference was found between the mean value of urinary calcium measured under basal conditions at the end of the “wash-out” phase, before starting water B intake (V3) (141.54 ± 104.40 mg/24 h) and the mean value of urinary calcium at the end of the three week period of intake of the same water (V4), which was equal to 129.61 ± 81.08 mg/24 h (Figure 2).

## 5. Discussion

According to the literature [28,31,32], our study, which enrolled young premenopausal women with low dietary calcium intake, further confirms the well-known bioavailability of calcium from calcium-rich mineral waters, which we measured through an indirect parameter. Indeed, after excluding values of 24 h urinary calcium of some participants from the statistical analysis according to the preliminary verification, we observed a significant increase in the levels of 24 h urinary calcium excretion (although within the normal range) at the end of the three week period of intake of the calcium-rich mineral water. Conversely, no increase in 24 h urinary calcium values was associated with the consumption of B water, which has a low calcium content. 

Urinary calcium excretion is the most relevant parameter for evaluating the systemic calcium balance. In the present study, the only variable introduced at the time of the analysis was the type of water ingested, with either low or high calcium content. Therefore, the variability observed in urinary calcium excretion can be considered as the indirect expression of increased intestinal calcium absorption.

It is known that ions other than calcium contained in mineral waters can influence urinary calcium excretion: sodium may promote it [42,43], while the effects of sulphate are not fully understood, as some authors reported a calciuric effect of sulphate, suggesting a better calcium balance with milk rather than with calcium and sulphate-rich waters [29], whereas others have not confirmed these data [25,28,44]. Mineral waters are defined as sodium-rich and sulphate-rich when the content of sodium and sulphate is > 200 mg/L, respectively [19,21]. Therefore, as the calcium-rich mineral water evaluated in our study is neither sodium-rich nor sulphate-rich (see Table 3), the increase in urinary calcium observed should not be attributed to the effect of the water ionic composition, but rather should be considered the result of the increased calcium load provided by a water rich in bioavailable calcium. 

Concerning the other laboratory parameters of mineral and bone metabolism, no influence of the content of calcium in the water was observed. These results can have a number of explanations. Our study population was composed of young pre-menopausal women with low dietary calcium intake, whereas two previous studies recruited young pre-menopausal women with sufficient dietary calcium intake [36] and post-menopausal women with low dietary calcium intake [35]. In particular, Wynn et al. [36] showed that a calcium-rich mineral water rich also in sulphate had no effect on serum PTH and on the serum bone resorption marker CTX in young premenopausal women with sufficient dietary calcium intake. On the other hand, a significant reduction of both serum PTH and CTX was observed in the same study population after the consumption of a bicarbonate-rich alkaline water with the same amount of calcium. The effect on PTH, and consequently on CTX, was therefore not attributed to the calcium content of water, but rather to the possible direct action of the alkaline load over the parathyroid cells. Calcium excretion significantly increased with the consumption of both waters [36]. The waters used in our study could not be assimilated to a sulphate-rich acid water or to a bicarbonate-rich alkaline water. The alkaline environment created by bicarbonate-rich waters is known to be, *per se*, beneficial for the reduction of bone resorption [19,44].

A study by Meunier et al. showed positive effects of calcium from a calcium and sulphate-rich water on bone biomarkers in a sample of 152 post-menopausal women with low dietary calcium intake (below 700 mg/day), showing a significant reduction of serum concentrations of PTH and markers of both bone resorption and formation after a six month period of consumption [35]. Our study recruited young women, and the period of observation was only three weeks. Moreover, the calcium-rich mineral water used in our study was not sulphate-rich. 

Our study did not evaluate acute effects of calcium-rich mineral water administration and acute effects on calcic metabolism could not be excluded, according to Guillemant et al. [40].

A limitation of this study is the small sample size. Thus, the absence of differences related to biochemical characteristics of mineral and bone metabolism reported in Table 4 could be due to low statistical power. Future research should increase the number of enrolled participants and the duration of the study to improve the statistical power. Another limitation is the duration of the study (nine weeks), as it could be hypothesized that a longer period of intervention might contribute to the appearance of the effects of calcium from a calcium-rich water on biochemical parameters of mineral and bone metabolism.

However, the positive aspect is that we recruited young women who were adherent and compliant to the study procedures (diet, regular intake of a prefixed amount of water, 24-h urine collection). Overall, the study design is innovative and suitable to assess the hypothesis we made.

## 6. Conclusions

Both calcium and water are essential elements for life, and their adequate intake is, therefore, fundamental for the maintenance of many body functions. Particularly, adequate dietary calcium intake is necessary for the maintenance of bone health, and calcium-rich mineral waters can represent a valid tool to reach this purpose. Indeed, calcium-rich mineral waters are a valuable source of highly bioavailable calcium with beneficial effects on both bone biomarkers and bone densitometric parameters.

Our study shows that a calcium-rich water can be a good source of dietary calcium in young women, providing a significant intake of bioavailable calcium, which we have indirectly evaluated through the measurement of 24-h urinary calcium. Therefore, our study confirms that calcium-rich mineral water is a valuable calorie-free nutritional source of highly bioavailable calcium, and that it can significantly contribute to achieving the daily requirements of this element. In our study the lack of the effects of calcium from calcium-rich water on bone biomarkers does not exclude the possibility that in a longer observation and in populations more prone to an increased bone remodeling status (i.e., post-menopausal women) this effect could be found. 

## Figures and Tables

**Table 1:** Recommended Dietary Allowance for Calcium (USDA: United States Department of Agriculture).

Life Stage Group	RDA/AI * (mg/day)
**Infants**	
0 to 6 months	* 200
6 to 12 months	* 260
**Children**	
1–3 years	700
4–8 years	1000
**Males/Females**	
9–13 years	1300
14–18 years	1300
19–30 years	1000
31–50 years	1000
51–70 years (males)	1000
51–70 years (females)	1200
>70 years	1200
**Pregnancy/Breastfeeding**	
14–18 years	1300
19–50 years	1000

* For infants 0 to 6 months and 6 to 12 months, these are Adequate Intakes (AI).

**Table 2:** Main characteristics of clinical studies investigating calcium bioavailability from mineral water and the effects of this ion on mineral and bone metabolism.

Scientific Article	Year of Publication	Number of Recruited Subjects	Aim of the Study	Endpoint
Halpern et al. [24]	1991	15 adult lactose-intolerant men	Bioavailability of calcium: calcium-rich water vs. milk	Tracer technique with calcium isotope
Heaney and Dowell [23]	1994	18 healthy women	Bioavailability of calcium: calcium-rich water vs. milk	Tracer technique with calcium isotope
Couzy et al. [25]	1995	9 healthy young adult women	Bioavailability of calcium: calcium- and sulphate-rich water vs. milk	Tracer technique with calcium isotope
Van Dokkum et al. [26]	1996	12 healthy young adult women	Bioavailability of calcium: calcium-rich water vs. dairy products	Tracer technique with calcium isotope
Cepollaro et al. [37]	1996	45 early postmenopausal women	Effects of calcium from calcium-rich water on densitometric parameters	Distal radius DXA
Wynckel et al. [27]	1997	12 students (8 males and 4 females)	Intestinal absorption of calcium from mineral waters with different calcium content	Tracer technique with calcium isotope
Costi et al. [38]	1999	255 pre- and post-menopausal women	Effects of calcium from calcium-rich water on densitometric parameters	Lumbar DXA
Aptel et al. [39]	1999	Data collection of 4434 women over 75 years from EPIDOS trial	Effects of calcium from drinking water on densitometric parameters	Femoral DXA
Guillemant et al. [40]	2000	12 healthy young male students	Acute effects (within 4 h) of calcium from calcium-rich water on biochemical parameters of bone metabolism	PTH, serum and urinary CTX
Bacciottini et al. [22]	2004	9 adult men + 9 pre-menopausal women + 9 post-menopausal women	Bioavailability of calcium: calcium-rich water vs. milk	Tracer technique with calcium isotope
Brandolini et al. [29]	2005	37 healthy young women	Bioavailability of calcium: calcium- and sulphate-rich water vs. milk	Urinary calcium
Meunier et al. [35]	2005	152 postmenopausal women with low dietary calcium intake	Effects of calcium from calcium-rich water on biochemical parameters of bone metabolism during a 6-month study period	PTH and biochemical markers of bone remodeling
Wynn et al. [36]	2009	30 healthy premenopausal women with sufficient dietary calcium intake	Effects of calcium-rich water on biochemical parameters of bone metabolism during a 4-week study period: calcium-rich alkaline water vs. calcium-rich acid water	PTH and serum CTX
Greupner et al. [28]	2017	21 healthy men and women	Bioavailability of calcium: 3 calcium-rich waters with different mineral content vs. milk vs. a calcium supplement	Serum and urinary calcium

Definition of abbreviations: DXA = dual-energy X-ray absorptiometry; PTH = parathyroid hormone; CTX = type 1 collagen cross-linked C-telopeptide.

**Table 3:** Composition of Uliveto water and B water.

	Uliveto Water	B Water
**Fixed Residue at 180 °C (mg/L)**	745	174.1
**pH**	5.8	7.56
**Bicarbonate (mg/L)**	570	182.1
**Chloride (mg/L)**	80	6.78
**Litium (mg/L)**	0.18	-
**Nitrate (mg/L)**	7.1	1.10
**Sodium (mg/L)**	67	4.13
**Silicon (mg/L)**	8.9	4.17
**Calcium (mg/L)**	173	57.63
**Fluoride (mg/l)**	1	0.13
**Magnesium (mg/l)**	25	3.23
**Potassium (mg/L)**	7.3	3.23
**Sulphate (mg/L)**	100	6.75
**Carbon Dioxide (CO_2_) (mg/L)**	1485	8.08
**Strontium (mg/L)**	-	0.23

**Table 4:** Biochemical characteristics of mineral and bone metabolism of the entire study group (*n* = 27) evaluated during a 9 week study period.

	V1	V2	*p*	V3	V4	*p*
**Serum Calcium (mg/dL)**	8.97 ± 0.35	8.94 ± 0.34	0.496	8.79 ± 0.36	8.93 ± 0.38	0.128
**Serum Phosphate (mg/dL)**	3.45 ± 0.48	3.51 ± 0.39	0.434	3.54 ± 0.91	3.47 ± 0.50	0.669
**Serum Magnesium (mg/dL)**	2.21 ± 0.13	2.17 ± 0.11	0.280	2.20 ± 0.20	2.19 ± 0.13	0.875
**Serum Total Proteins (g/dL)**	7.40 ± 0.32	7.31 ± 0.35	0.212	7.27 ± 0.29	7.31 ± 0.27	0.323
**Serum PTH (pmol/L)**	5.71 ± 3.04	5.67 ± 1.98	0.956	5.54 ± 2.09	5.41 ± 1.83	0.720
**Serum 25(OH)vitaminD (ng/mL)**	21.74 ± 7.98	21.95 ± 6.92	0.838	19.84 ± 6.32	20.97 ± 4.94	0.299
**Serum BALP (mcg/L)**	15.31 ± 4.20	15.60 ± 4.16	0.149	14.93 ± 4.14	14.75 ± 4.53	0.697
**Serum Beta-CTX (ng/mL)**	0.310 ± 0.121	0.319 ± 0.120	0.630	0.335 ± 0.132	0.337 ± 0.127	0.912
**Urinary Phosphate (mg/24 h)**	690.31 ± 427.30	600.18 ± 485.04	0.580	565.78 ± 307.40	531.14 ± 321.65	0.538

Data were expressed as mean ± SD. Statistical analysis was performed using Student’s *t*-test. For the definition of the abbreviations see the text.

## Abbreviations

PTH	parathyroid hormone
1,25(OH)_2_D	1,25-dihydroxyvitamin D
FGF23	Fibroblast Growth Factor 23
VDR	vitamin D receptor
CTX	type 1 collagen cross-linked C-telopeptide
BALP	bone alkaline phosphatase
